# Perinatal Mortality

**Published:** 1958-10

**Authors:** Thomas E. Oppé

**Affiliations:** Lecturer in Child Health, University of Bristol


### PERINATAL MORTALITY

BY

THOMAS E. OPPÉ, M.B., M.R.C.P.

*Lecturer in Child Health, University of Bristol*

Sophisticated peoples have a low fertility and great concern for the welfare of their children. Their low birth rate is due to deliberate family limitation; besides planned parenthood, their desire for better living standards leads to emancipation of women, and later marriages. A century ago in Britain, when conditions of infant life were appalling, no less than 15 out of every 100 live born infants did not live to see their first birthday: at the same time, each year every 100 women of child-bearing age were producing 15 babies. Now, 97 per cent of infants survive their first year, but our potential mothers give birth at the annual rate of only 7.3 per cent.

The immense gain in infant survival has compensated for the losses due to lowered fertility. However, the point has now almost been reached where no further decreases in infant and child mortality are possible, and even if the present low mortalities were reduced to zero the increase in population would be negligible. If a fall in population is to be avoided it is necessary either to reduce reproductive wastage at an earlier stage or to create conditions favourable for increased fertility. The ideal population would combine a low infant death rate with high fertility, which is most nearly achieved by the U.S.A. where the population is increasing at the rate of 2 per cent, per annum. Although estimates of reproductive wastage are imprecise it is probable that there are each year in Britain some 250,000 miscarriages; many of these are abnormal foetuses, others are unwanted pregnancies but apart from them a great number of potential lives are lost. Once the foetus has reached viability (at twenty-eight weeks) its fate is more accurately known; the hazards of late pregnancy and delivery account for 26,000 deaths each year, a casualty list twice that of carcinoma of the lung and five times that due to fatal road accidents.

*Perinatal Mortality Rate:*

Death of the infant at about the time of birth is described as "perinatal". In Britain perinatal mortality is defined as "the combination of stillbirths and first-week neonatal deaths". If these are expressed as deaths per 1,000 total births the "Perinatal Mortality Rate" (P.M.R.) is obtained. This P.M.R. is a useful concept because it emphasises the episodic nature of birth in the life of the individual from conception to dissolution, and it eliminates the often arbitrary distinction between stillbirth and early neonatal death. However, the British definition of P.M.R. is neither recognised nor used widely. One objection is that lethal congenital malformations, some intra-uterine deaths and some post-natal deaths are not directly associated with the process of birth. Moreover, it is often inconvenient to present statistics sharply drawn at the seventh post-natal day.

In Britain the P.M.R. is about thirty-seven per 1,000 total births. Twenty-three of these are stillbirths and fourteen are early neonatal deaths. Unlike the post-natal death rate (i.e. deaths occurring between the fourth week and the end of the first year of life) the P.M.R. has not greatly altered in recent years, and for ten years the stillbirth rate has remained the same. Efforts to improve these figures are not a mere academic task, for this loss of life is ill-spared by the community and it also represents much personal disappointment and family tragedy. It is not always true to say that further pregnancies can make good an unsuccessful one, for too often it is the precious child that is lost, or subsequent fertility is impaired through physical causes, or by suppression of maternal feelings and desires.

The causes of perinatal mortality are diverse. Problems of maternal, placental and foetal pathology are inextricably woven into the fabric of genetic, economic and social factors. Not all pregnant women avail themselves of the present obstetrical services and skill and there are varying standards of medical care. All aspects of the problem must be investigated if it is to be solved. Research in perinatal mortality has followed three main paths. (1) Analysis and correlation of the data obtained from death certificates. (2) Clinico-pathological studies of individual cases has yielded fresh facts about pathological conditions causing perinatal death and their prevention and treatment. (3) the study of individual factors implicated in perinatal mortality have helped to find the most satisfactory methods of obstetric and paediatric care.

*Statistical Approach:*

The statistical analysis of the notifications of stillbirths and neonatal deaths has been most rewarding in defining the extent of the problem and the areas which are succeeding or failing to deal with it. Important correlations have also been made with other statistical data mainly of a socio-economic kind. Thus perinatal deaths are not uniformly distributed either between or within nations. For instance, the perinatal mortality of Wigan is twice that of Oxford, while Bristol comes close to the national average[1]. Interpretation of this statistical information is of course limited by the uncertainty of the data provided by birth notifications and death certificates. If mortality data are to be compared on an international scale, both terminology and presentation of facts must become uniform. At present this is not so, but the deliberations of the Statistical Commission of the United Nations and the W.H.O. Committee on Health Statistics should secure common agreement on these matters. It is not only statistics on the grand scale which make valuable contributions. Such relatively modest surveys as those carried out in Bristol[2] and the S.W. Region[3] have produced results of more than local interest. Both these surveys showed that in an economically-thriving population there were no significant differences in perinatal mortality between one social class and another. In neither of these surveys could poor medical care be held responsible for more than a small proportion of deaths. In the Bristol series, 8 per cent of deaths might have been prevented by better co-operation of the patient, by more competent obstetric management or by more adequate services. On the other hand thirty-five per cent of perinatal deaths in New York City[4] were recently held preventable on the grounds of inadequate prenatal care, error in medical judgment, error in medical technique, family at fault, unqualified medical attendant or inadequate paediatric care. A direct comparison between these two observations is possibly not valid, but we must not be complacent about our own mortality figures. Whatever the true position it is important that our maternity services should be based on knowledge rather than prejudice, whimsy and politics.

*Clinico-Pathological Study:*

While statistical information is vital for the demographer and the administrator it is anathema to many clinicians. They are concerned with the management of individual pregnancies and not the manipulation of masses of gravid units. The clinical obstetrician and paediatrician have much to learn from the detailed clinico-pathological study of many individual infant deaths. Such work can only take place in maternity departments which deal with large numbers of cases and command expert clinical and pathological services. Maternity units of this quality usually admit cases selected on medical or social grounds and thus tend to deal with a high proportion of abnormal midwifery. Mortality figures from such a source do not reflect the over-all problem of perinatal mortality, but they do indicate clearly the types of lesion encountered. The most detailed recent study of perinatal mortality undertaken in Britain is that of Bound, Butler and Spector[5] from University College Hospital. They divided deaths into three main groups: antepartum, intrapartum and postpartum deaths. Antepartum deaths were further differentiated into those, without evidence of asphyxia (usually macerated) and fresh stillbirths with signs of asphyxia. The former were most often due to maternal toxaemia, high maternal age, small antepartum haemorrhages, maternal diabetes or rhesus incompatibility. Acute asphyxial deaths had similar maternal histories, but catastrophes such as severe antepartum haemorrhage or prolapse of the cord were also frequently found. Intrapartum deaths were anoxic or traumatic in origin and were associated with prolonged or complicated labours. Post-partum deaths occur against an overwhelming background of prematurity, although birth trauma and infection are not uncommon.

For several years perinatal mortality has been closely studied at Southmead Hospital, Bristol. Dr. Norman J. Brown[6] has kindly given me permission to publish the results of his careful post-mortem investigations on perinatal deaths. In the three years 1955–57, there have been 739 stillbirths and neonatal deaths at Southmead Hospital, almost all of which fall within the category of Perinatal deaths. Of these, 59 per cent were stillbirths, 26 per cent neonatal deaths in premature infants and 15 per cent neonatal deaths in full-term infants. In 648 deaths (88 per cent) the cause was accurately determined on a basis of clinical and pathological studies. Lethal congenital malformations (the majority being anencephaly or spina bifida) were responsible for 164 deaths (22 per cent). While it is not intended to minimise the importance of congenital malformations as a cause of reproductive failure, they are not strictly relevant to the present discussion. Whether congenital malformations are due to genetic or environmental causes—or the facilitation of one by the other, it is generally accepted that with rare exceptions they are determined during the first three months of pregnancy and are thus not part of the hazards of late pregnancy. If these deaths are excluded as not resulting primarily from perinatal causes there remain 494 autopsies the findings of which are available. Excluding lethal congenital malformations altogether, we found 270 stillbirths (55 per cent) 153 premature neonatal deaths (33 per cent) and 53 full-term deaths (12 per cent). Pre-eclamptic toxaemia was the pre-eminent cause of perinatal death. Maternal toxaemia or hypertension was associated with 20 per cent of the stillbirths and was responsible for the early onset, or induction of labour in 21 per cent of the premature deaths. Other major causes of stillbirths were ante-partum haemorrhage and placental infarction not due to toxaemia. Birth trauma and acute intrapartum asphyxia was responsible for many fresh stillbirths. Postmaturity, maternal diabetes and erythroblastosis also appeared as causes of foetal death.

*Effects of Prematurity:*

Consideration of the neonatal deaths show how great is the problem of prematurity. While only 6 or 7 per cent of viable pregnancies terminate prematurely, these premature deliveries account for 74 per cent of all neonatal deaths. Most of the deaths (77) in premature infants are associated with pulmonary inadequacy. At autopsy, atelectasis or gross immaturity of the lungs is found. Intra-ventricular cerebral haemorrhage, probably asphyxial in nature, is frequently associated with pulmonary atelectasis; in a few cases intra-cranial haemorrhage is obviously traumatic in origin. The clinical aspects of these premature deaths are perplexing. Pulmonary inadequacy is usually manifest not at birth but a few hours after when tachypnoea and grunting respirations appear; the condition worsens with retractions of the ribs or sternum, intense dyspnoea, cyanosis and apnoeic attacks; often the infant becomes, and remains, oedematous. At autopsy so-called "hyaline membranes" are frequently found distributed around the walls of the alveolar ducts which are surrounded by atelectatic alveoli. Apart from a consistent relationship with prematurity, infants of diabetic mothers and rarely full-term infants born by Caesarean section, no fundamental facts are known about the aetiology of this pulmonary syndrome. Because treatment of this condition is ineffectual and the proper management of prematurity is exceedingly expensive, prevention of prematurity is far more important than its cure.

Few live-born full-term infants without serious congenital malformations die.

Trauma, infection and erythroblastosis are the conditions most likely to cause death. Birth trauma might be prevented by alternative methods of delivery, infection is now an inseparable hazard of hospital midwifery, and erythroblastosis should cause very few neonatal deaths were ante-natal prediction and well-timed delivery always practised[7].

*Study of Special Aetiological Factors:*

The third investigational approach to perinatal mortality is the study of all the factors concerned or believed to be concerned in diminishing the infant's chance of survival. In a short review it is impossible to survey all the reports which are relevant to the concatenation of events which may lead to a dead baby. Amongst these must be included any deviation from the normal in the anatomy or physiology of the mother; the adequacy of the placenta and the integrity of the umbilical pathway; the capacity of the pelvis and the forces of labour; the size and maturity of the foetus, its presentation or organisation, all influence the outcome of pregnancy. Interference with natural parturition by drugs or instruments may do great good but, used unwisely, may be harmful. The majority of these factors have been analysed and we believe that modern obstetric and paediatric practice is based on scientific facts; however, much has yet to be learned and many of our traditional procedures are over-due for critical review.

Probably the largest single factor contributing to perinatal mortality is pre-eclamptic toxaemia. Its aetiology still remains a mystery in spite of recent research. Much of its baneful effects on the foetus can be minimised by good ante-natal care; this has been clearly shown by Hughes[8] who has by empirical measures greatly reduced the incidence of severe toxaemia and cut down the premature neonatal death rate. The recent work of Robinson[9] suggests that salt added to the diet of the pregnant woman not only prevents toxaemia but may reduce by almost half the perinatal death rate; if this is confirmed it will revolutionise our treatment.

Another hotly debated factor in perinatal death is postmaturity. All the pontifications of the pundits indicate that the practitioner need not be unduly alarmed. Provided that elderly primiparae and women with the slightest toxaemia or hypertension are not allowed to progress beyond term it seems that interference is probably unjustified.

The management of prematurity is also undergoing revision. When should the umbilical cord be clamped? We were told some years ago to delay clamping it until pulsations ceased because the premature baby needed the extra blood from the placenta to prevent subsequent anaemia. Later we were then told that the baby had too many red cells and that by delayed clamping we were contributing to neonatal jaundice. Now we no longer believe that neonatal jaundice is due to excess haemolysis (apart from blood incompatibility) and once more we must let the placental blood drain into the baby lest there be inadequate blood volume to erect the lungs[10]. Heat, humidity and oxygen were once the major therapeutic weapons for all the ills of the premature infant—for did not babies die of anoxia and did they not emerge from a hot, wet environment? The babies which survived this enthusiastic treatment with oxygen often developed retrolental fibroplasia and became blind. To-day it is reasonably suggested that premature infants should be nursed in artificially low concentrations of oxygen[11] and that hypothermia is beneficial. The last word has not been written on any aspect of pregnancy, parturition or paediatric care and only by continuing research will all the truth be known.

The la est method of acquiring knowledge about perinatal mortality is the large-scale socio-medical enquiry. This technique, derived from the discipline of Social Medicine, should prove useful in the field of perinatal mortality. For one week in March this year a detailed questionnaire was completed on every birth in Britain and covered almost every relevant social and medical factor. As many perinatal deaths as possible were referred to selected pathologists for detailed examination and the results will be correlated with the obstetric and paediatric histories. This survey is being carried out under the auspices of the National Birthday Trust; it is hoped the facts so found will show those areas where maternity services might be improved and will indicate the fields where intensive research is needed. The survey comes at an opportune time, not only because of the present interest in perinatal death but also because a revision of maternity services is now being considered. This revision may well affect the professional lives of many family doctors and may alter the whole structure of the profession of midwifery.

I wish to acknowledge the help given by the Obstetricians and Paediatricians at Southmead Hospital, Bristol and especially the kindness of Dr. N. J. Brown who has made available pathological material and reports.

### REFERENCES

[1] *Registrar-General's Statistical Review of England and Wales.* (1954) London, H.M.S.O.
[2] *Stillbirths and Neonatal Deaths in Bristol.* Southmead General Hospital Management Committee. Bristol, 1952.
[4] *Maternity Service.* South Western Regional Hospital Board. Bristol, 1956.
[4] *Perinatal Mortality in New York City.* Commonwealth Fund. Harvard University Press. Cambridge, Mass. 1957.
[5] Bound, J. P., Butler, N. R. and Spector, W. G. 1956. *Brit. Med. J.*, **2,** 1191 and 1260.
[6] Brown, N. J. Personal communication.
[7] Walker, W. and Mollison, P. L. 1957. *Lancet,* **1,** 1309.
[8] Hughes, T. Dixon. 1956. *Med. J. Aust.*, **2,** 48.
[9] Robinson, Margaret. 1958. *Lancet,* **1,** 178.
[10] Bonham Carter, R. E. 1957. ibid., **1,** 1292.
[11] Sjostedt, S. and Rooth, G. 1957. *Arch. Dis. Child.*, **32,** 3917.

